# Unraveling the mechanisms of slack channel mutations in epileptic disorders

**DOI:** 10.1186/s42494-026-00246-6

**Published:** 2026-03-02

**Authors:** Yue Wei, Aqeela Zahra, Qun Wang, Jianping Wu

**Affiliations:** 1 Department of Pharmacology, School of Basic Medical Sciences, Hubei University of Medicine, Shiyan, 440070 China; 2 School of Chemistry, Chemical Engineering and Life Sciences, Wuhan University of Technology, Wuhan, 430070 China; 3 Beijing Tiantan Hospital, Capital Medical University, Beijing, 10070 China; 4 China National Clinical Research Center for Neurological Diseases, Beijing, 10070 China

**Keywords:** Slack channel, *KCNT1*, Epilepsy, Fragile X syndrome, Anxiety disorder

## Abstract

Sodium-activated potassium channels (K_Na_) are extensively expressed throughout the central nervous system (CNS) and play a pivotal role in modulating neuronal excitability. The K_Na_ achieve this by regulating the threshold for action potential initiation and the magnitude of post-hyperpolarization. The *KCNT1* gene encodes the α subunit of K_Na_, also known as Slack channel, is crucial for governing the membrane excitability of neurons. Mutations in the *KCNT1* gene impair the function of these potassium channels, leading to seizures and a spectrum of epileptic disorders. These conditions are often intractable and can range in severity from moderate to profound. This article delves into the subject of Slack channel detailing their architecture, physiological functions, distribution, and expression patterns, as well as their essential role in the nervous system. Additionally, we address the topic of epilepsy resulting from mutations in the *KCNT1* gene and the therapeutic strategies currently available for managing these challenging conditions.

## Background

Epilepsy, a prevalent neurological disorder, is characterized as a chronic brain condition marked by recurrent, stereotyped seizures [1, 2]. The abrupt and concurrent abnormal firing of numerous neurons leads to temporary dysfunction of the central nervous system (CNS). Current research indicates that genetic variations in ion channels account for approximately one-third of the known single-gene causes of epilepsy [3].The neural network incorporates various regulatory mechanisms that are pivotal in inhibiting or ceasing the continuous firing of neurons, notably through after-hyperpolarizations (AHPs). AHPs is an outward potassium current initiated by an action potential (AP), and its amplitude and duration critically influence the occurrence and timing of subsequent APs, thereby regulating the frequency, pattern, and precision of neuronal activity [4, 5]. During an AP, intracellular sodium concentrations increase, leading to the activation of sodium-activated potassium channels (K_Na_ channels). K_Na_ activation in primary excitatory neurons reduces excitability by extending the refractory period and inhibiting excessive firing, consequently preserving network stability. K_Na_ channels similarly regulate the firing patterns of interneurons, thereby influencing inhibitory control within neural circuits. The activation of these channels at the microcircuit level causing neuronal ensemble excitability and synchronization, thereby impacting information processing and network oscillations. K_Na_ channel activity following AP is essential for maintaining the balance between excitation and inhibition, thereby ensuring proper neural function and mitigating disorders associated with hyperexcitability [6].

K_Na_ are composed of subunits encoded by two mammalian genes, known as *KCNT1* and *KCNT2*. The *KCNT1* gene encodes the α-subunit of K_Na_ channels, where its activation requires elevated intracellular sodium concentrations ([Na^+^]_i_, 50 ~ 70 mM). K_Na_ channels may be structurally or functionally coupled with voltage-dependent sodium channels (Nav), NMDA, and AMPA receptors. During spontaneous or bursting neuronal activity, Na^+^ influx can lead to localized transient increases in [Na^+^]_i_ (> 100 mM) and activate the associated K_Na_ channels. Mutations in the *KCNT1* gene are strongly linked to various forms of epilepsy, including epilepsy with migrating focal seizures of infancy (EIMFS), autosomal dominant nocturnal frontal lobe epilepsy (ADNFLE), and other early-onset epileptic encephalopathies (EOEE) [4]. Epilepsy associated with *KCNT1* gene mutations is predominantly autosomal dominant and phenotypic, often presenting with focal seizures, pronounced clinical features, and early onset. These cases are frequently accompanied by mental and behavioral abnormalities and congenital developmental issues, with a poor response to antiseizure medications (ASMs). Early diagnosis is crucial for targeted treatment and prognostic evaluation.

Understanding the mechanisms of epilepsy is essential for effective treatment. While the exact mechanism remains elusive, it appears that excessive and coordinated neural activity could play a significant role in epilepsy [5–7]. This article examines the role of ion channels, particularly the *KCNT1* gene and its potassium channels, in epilepsy pathogenesis. The objective is to highlight the genetics of epilepsy, specifically how mutations affect neuronal excitability and how they affect various types of epilepsy.

## Structure and function of Slack channels

### Structure

**Fig. 1:**
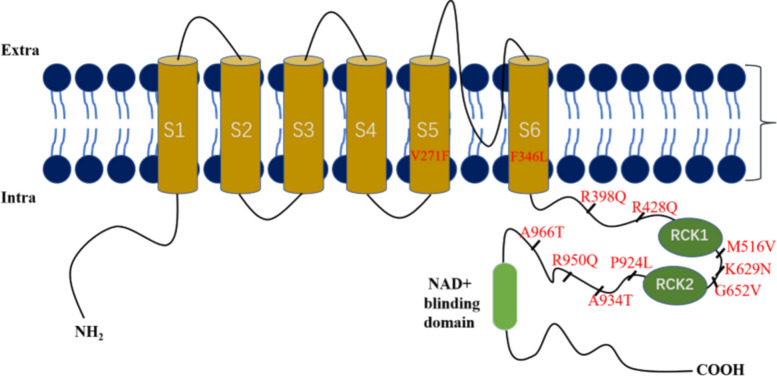
Slack channel topology. Shows an S1-S4 transmembrane domain; A hole-lining ring formed by S5 and S6; long C-terminal containing two RCK domains and a NAD + binding domain; Site of mutation; Partial mutation sites

The Slack channels are encoded by the *KCNT1* gene, which is located on chromosome 9q34.3 in humans, chromosome 2 in mice, and chromosome 3 in rats [8]. These channels exhibit an exceptionally high conductance of approximately 180 pS in mammals cells [9]. The Slack channel subunit comprises six hydrophobic transmembrane segments (S1-S6), with a hole-lining ring formed between segments S5 and S6 [10]. Additionally, it features a large intracellular C-terminal domain (CTD) that contains two potassium conductance regulation (RCK) domains and a niacinamide adenine dinucleotide (NAD^+^) binding domain, as depicted in Fig. 1 [11–13].

### Function

Unlike the voltage-gated potassium channel family, Slack channels do not have a voltage-sensing domain (VSD) and lack charged residues in their S4 segments [14]. Consequently, the exact mechanism underlying their voltage sensitivity remains unclear. Slack channels exhibit a high dependency on Na^+^ and a less sensitivity to Cl^−^ [15]. Mutational analysis has linked the Na^+^ sensitivity of Slack channels to the RCK domain [16, 17]. An increase in Na^+^ concentration prompts a conformational change in the RCK domain, which forms a ring on the cytoplasmic surface, potentially exposing the electrostatic channel pores. However, the precise mechanism has yet to be delineated. The NAD^+^ binding domain then modulates the channel’s requirement for Na^+^ by altering NAD^+^ concentrations. The formation of a heterotetramer by SLACK and SLICK at a 1:1 molar ratio results in a (SLACK)(SLICK) complex. Biochemical and structural research confirm this stoichiometry's symmetric construction. Both proteins' conserved C-terminal coiled-coil domains essentially assemble them. These coiled-coil domains promote heteromeric connections and stable heterotetramers. The coiled-coil region provides the structural interface for oligomerization in heteromeric complexes, making it the assembly domain. Many N-terminal domains are important in targeting or regulatory interactions but not tetramer formation [18, 19]. The actual function of K_Na_ channels depends on their composition, distribution, cellular and subcellular localization [20]. At the same time, K_Na_ channels are the basis for the generation of K_Na_ currents. This K_Na_ current was initially observed in avian trigeminal ganglion neurons in 1985 [21]. In the standard electrophysiological contexts, Slack channels regulate neuronal excitability by regulating potassium transport and membrane potential changes. During the AP, Slack channel activation induces rapid potassium efflux, driving membrane potential repolarization, terminating neuronal excitation and avoiding functional disorder. In the resting state, the channel opening rate is reduced, which limits potassium efflux and maintains membrane potential homeostasis, thus ensuring the normal function of neurons [22–24].

## Distribution and expression of Slack channels

Studies have found that *KCNT1* gene, which encodes Slack channel, is expressed in the whole life cycle of the human body. It is involved in the early development of nerve cells in the embryonic period, contributes to the maturation of neurons and the construction of neural networks in the neonatal period, and continues to be expressed in nerve cells after adulthood to maintain neuronal excitability and nervous system homeostasis. Meanwhile, previous studies found that Slack channels were highly expressed in the CNS of mammals, including crucial regions such as the olfactory bulb, hippocampus, amygdala, lateral septal nucleus, vestibular system, frontal cortex, thalamus, and other brain areas. Additionally, as illustrated in Table 1, the expression of Slack channels has been observed in mammalian hearts. Overall, the Table 1 outlines many interactions and activities within the neurological and circulatory systems, emphasizing how certain actions may lead to distinct physiological results.

**Table 1:** Distribution and physiological function of Slack channels

System	Location	Action process	Action result	Reference
Nervous system	BLA	BLA-vHPC projections	Regulate anxious behavior	[25]
	DRG	Neuronal excitability	Nociceptive behavior	[26]
	DRG	Depolarization-elicited AP	Itch	[27]
	MNTB	Accuracy of AP time	Auditory	[28]
	OB	Provide a major outward current	Olfactory	[29]
	VANs Cortex	AHP Reduce the excitability of cortical GABAergic neurons	Regulate the body's balance Seizures of epilepsy	[30] [31]
Circulatory system	Heart	Gated by Na^+^	Heart disease	[32]

*Abbreviations*: *DRG* BLA; Basolateral amygdala, *MNTB* Dorsal root ganglion, *OB* Medial nucleus of the trapezoid body, *VANs* olfactory bulb, *AP* Vestibular afferent neurons, *AHP* Action potential After-hyperpolarization

## The role of Slack channels in neurological disorders

### Fragile X syndrome (FXS)

*KCNT1* gene encodes Slack channel that modulate firing patterns and neuronal excitability across various neuron types and may have implications in fragile X syndrome (FXS) [33–35]. The fragile X mental retardation protein (FMRP) is a critical neuronal RNA-binding protein associated with numerous ribosomes, suggesting a pivotal role in the regulation of neuronal RNA translation. Nonetheless, the precise RNA targets and the underlying mechanism of FMRP’s action remain elusive [36]. A hallmark of FXS is neuronal hyperexcitability, which gives rise to a spectrum of symptoms including moderate to severe intellectual disability, developmental delays, attention deficit hyperactivity disorder (ADHD), emotional lability with anxiety, obsessive–compulsive disorder, and autistic-like behaviors [37]. Additionally, individuals with FXS may exhibit poor motor coordination, heightened sensitivity to sensory stimuli, and an elevated risk of epilepsy [38].

Previous research has demonstrated that FMRP interacts with the cytoplasmic C-terminal domain of the Slack, thereby enhancing their activity [39]. These results imply that Slack channel activity may serve as a critical bridge connecting neuronal firing patterns to alterations in protein translation [40]. Recently, the distinct social behavior deficits observed in Slack channel knockout animals have been noted to differ from those in FMRP knockout mice [33]. Although experimental evidence suggests that FMRP interacts with Slack channels and influences their activity, this does not imply that such interaction directly results in a clear effect on overall neuronal excitability in physiological conditions. Consequently, the precise role of Slack channels in FXS is still unclear and necessitates additional investigation.

### Epilepsy

The primary form of epilepsy associated with *KCNT1* is caused by gain-of-function (GOF) mutations in the channel proteins, as reported in studies [41, 42]. These mutations are predominantly found within and around the channel’s structural domains, with a particular concentration in the RCK^−^ and NAD^+^-binding domains, which are considered mutation hotspots. Moreover, pathogenic variants have been detected in the pore-forming region that spans between the S5 and S6 segments. Research to date indicates that the position of mutations within the channel’s architecture may be linked to the clinical phenotypes observed [43]. For example, the G288S mutation located on S5 is associated with autosomal dominant nocturnal frontal lobe epilepsy (ADNFLE), while the R398Q mutation situated on RCK1 is linked to epilepsy of infancy with migrating focal seizures (EIMFS) [44]. Despite these findings, the precise correlation between the sites of mutations and the clinical manifestations in patients exhibiting diverse epilepsy phenotypes is not yet fully understood.

Regarding the mechanisms by which GOF mutations in the Slack channel lead to excessive channel activity, two primary hypotheses have been proposed: increased sensitivity to sodium ions and an elevation in the channel’s maximum open probability [45]. Various hypotheses have been suggested to explain how GOF mutations precipitate seizures. One proposal is that in excitatory neurons, APs experience more rapid repolarization while sodium channel inactivation is minimized, thereby increasing the frequency of AP firing [31]. Another study suggests that in inhibitory interneurons, a decrease in membrane excitability and enhanced adaptive AP firing lead to disinhibition mediated by GABA, which in turn raises network excitability [46]. Additionally, alterations in AP firing patterns during neuronal development can result in modified synaptic connectivity, contributing to the formation of hyperexcitable networks and epileptogenic foci [47]. Furthermore, aside from directly impacting neuronal excitability, these mutants may disrupt intracellular signaling pathways that involve FMRP and its dependent protein translation [40]. The role of Slack channel mutations in epilepsy, the neural circuits involved, and the underlying pathogenic mechanisms all warrant additional research.

#### Associated epilepsy

A correlation has been noted between early-onset epileptic encephalopathy and functional enhancement mutations in the Slack channel subunits, leading to significant clinical variability in the phenotypes of patients affected by this condition. This variability encompasses disorders such as EIMFS, ADNFLE, and EOEE (early-onset epileptic encephalopathy). It is of particular interest that identical mutation sites can result in different phenotypes. For example, mutations p.G288S, p.R398Q, and p.A934T have been identified in both EIMFS and ADNFLE, as detailed in Table 2.

**Table 2:** Pathogenic Slack channel variant and clinical features

Pathogenic mutations	Location	Associated phenotype(s)	Type of mutation	References
R209C	S3 domain	LGS	GOF	[48]
A259D	S4-5 linker	EIMFS	GOF	[49]
Q270E	S5 domain	EIMFS	GOF	[50]
V271F	S5 domain	EIMFS	GOF	[51]
L274I	S5 domain	EIMFS	GOF	[4]
G288S	S5 domain	ADNFLE EIMFS	GOF	[52]
F346L	S6 domain	EIMFS	GOF	[53]
R398Q	RCK1 domain	ADNFLE EIMFS	GOF	[43]
R428Q	RCK1 domain	EIMFS	GOF	[54]
M516V	RCK1 domain	EIMFS	GOF	[55]
K629N	RCK2 domain	EIMFS	GOF	[56]
G652V	RCK2 domain	WS	GOF	[57]
Y796H	NAD^+^ blinding domain	ADNFLE	GOF	[58]
M896K	NAD^+^ blinding domain	EIMFS	GOF	[51]
P924L	C-terminus	EIMFS	GOF	[53]
A934T	C-terminus	ADNFLE EIMFS	GOF	[59, 60]
R950Q	C-terminus	EIMFS	GOF	[61]
A966T	C-terminus	OS	GOF	[62]

*Abbreviations*: *LGS* Lennox-Gastaut syndrome, *EIMFS* Epilepsy with migrating focal seizures, *ADNFLE* Autosomal dominant nocturnal frontal epilepsy, *WS* West syndrome, *OS* Ohtahara syndrome

#### EIMFS

Early infantile migratory focal seizures, initially described by Coppola et al. in 1995 [63], are a form of rare developmental and epileptic encephalopathy (DEE) that was officially recognized by the International League Against Epilepsy (ILAE) in 2010. Neuroimaging studies usually show no specific structural abnormalities. Some patients may have hypometabolism during the interictal period, and may have functional changes such as increased regional cerebral blood flow during the ictal period [64]. EIMFS is genetically linked to GOF mutations in the *KCNT1* gene [65]. Approximately 40% of EIMFS cases are attributable to *KCNT1* mutations, with recurrent pathogenic variants clustered at residues p.G288S, p.R428Q, and p.A934T [66]. Numerous case studies and reports have highlighted *KCNT1* mutations in individuals with severe EIMFS, marked by early-onset, refractory seizures and developmental delay [67]. While most *KCNT1* mutations arise de novo, the disorder exhibits genetic heterogeneity, with additional implicated genes including *SCN1A*, *SCN2A*, *SCN8A*, *SLC25A22*, *SLC12A5*, *TBC1D24*, and *PLCB1*. The prognosis is generally poor across all etiologies, with *KCNT1*-related cases demonstrating particularly severe outcomes characterized by pharmacoresistant seizures and profound neurodevelopmental stagnation.

#### ADNFLE

Sleep-related hypermotor epilepsy (SHE) is a form of focal epilepsy characterized by “hypermobility” often accompanied by asymmetric tonic or dystonic posturing. This condition is heterogeneous, encompassing both sporadic and familial cases, with ADNFLE being the most prevalent familial form of SHE [68, 69]. Mutations have been associated with ADNFLE, often manifesting as nocturnal frontal lobe seizures. Neuroimaging studies, such as CT or MRI of the head, mostly appeared normal. Rarely, nonspecific changes such as mild atrophy of the frontal lobes or mild abnormalities of the white matter may be found [70]. The etiology for most patients with SHE remains unknown, although a subset with familial SHE is linked to genetic mutations [71]. In 1994, Scheffer and colleagues [72] reported on a large Australian family with ADNFLE and identified the *CHRNA4* gene, which encodes the α4 subunit of the neuronal nicotinic acetylcholine receptor (nAChR), as the first confirmed gene causing familial SHE. Subsequent research has detailed mutations in other nAChR subunit genes, such as *CHRNA2* and *CHRNB2* [73]. Mutations in the *CHRNA2* gene are more frequently associated with ADNFLE and may result in a loss of functional phenotype. In addition to mutations in the neuronal nAChR subunits, mutations in the *KCNT1* gene have also been linked to ADNFLE cases [74]. Patients with *KCNT1* mutations tend to exhibit more severe phenotypes, including an earlier age of onset, higher rates of drug resistance, and an increased incidence of intellectual disability and psychobehavioral disorders.

#### EOEE

Research indicates that variations in the *KCNT1* gene can lead to EOEE, with the exception of EIMFS and ADNFLE. This encompasses a range of conditions, including West syndrome (WS), Ohtahara syndrome (OS), early-onset myoclonic encephalopathy (EME), and Lennox-Gastaut syndrome (LGS), among other similar disorders. EOEE is characterized by early onset and significant developmental impairment [66]. Neuroimaging findings were diverse. Some patients may have abnormal brain structural development, such as cortical dysplasia, leukodystrophy, and brain atrophy. Some patients may have abnormal brain metabolism, such as decreased glucose metabolism in local brain tissue by positron emission tomography (PET) examination.

## Treatments

Each treatment method of epilepsy has its advantages and disadvantages, which together constitute a multi-dimensional treatment system. The specific treatment methods are shown in Table 3.

**Table 3:** Comparing clinical and experimental treatments based on evidence of Slack channel

Type	Treatment	Clinical experimental	Evidence type	Advantages	Disadvantage
Channel blocker	Quinidine	Both	Case report/Randomized crossover trial	Reduce seizure	Variable response (arrhythmia) [75]
Small molecule novel Slack inhibitor	VU0606170	Both	In vitro electrophysiology; cultured neurons; mouse/cortex neuron models	Reduces neuronal hyperexcitability; potential for fewer off-target effects	Not yet tested in humans; pharmacokinetics/safety unknown [76]
ASO	*KCNT1*-targeted ASO	Preclinical expriment	Mouse model GOF (*KCNT1) *mutation	Reduce pathogenic overexpression	Unclear [77]
Dietary	Ketogenic diet	Clinical	Randomized controlled trial/Cohort studies/case reports in specific seizures	Reduce seizure	Difficult to maintain in (acidosis, kidney stones etc.) [78]
Surgery	Focal resection lobectomy	Clinical	Established infocal epilepsies	Can be curative or drastically reduce seizure	Neurological deficits, multifocal diffuse [79]
Behavioral complementary therapies	Neural feedback music therapy, yoga, etc	Both	Case report	Low risk, non-invasive	Likely modest variable response [80]
Other neuromodulation	Deep brain stimulation (DBS); transcranial magnetic stimulation (TMS)	Both	Some trials/case series	Potential additional tool	Cost; invasiveness,limited data; unclear [81]

### Inhibitors

Conventional treatments for *KCNT1*-related epilepsy generally offer only temporary relief from symptoms, and the majority of cases show resistance to pharmacological interventions. Quinidine, a nonspecific potassium channel blocker, has been suggested as a promising treatment option due to its capacity to regulate abnormal channel activity. Preclinical studies have shown that quinidine can inhibit both normal and disease-causing Slack channels in various expression systems, such as Xenopus oocytes and mammalian cells [58, 61]. However, its clinical efficacy is marked by substantial heterogeneity. An international multicenter trial reported a ≥ 50% reduction in seizure frequency in 45% of quinidine-treated patients with *KCNT1* mutations [4]. In contrast, a retrospective cohort study with 20 participants found similar effectiveness in only 20% of patients, with fleeting improvements limited to those carrying mutations near the RCK2 domain in the carboxyl-terminal NADP-binding region [52]. Further complicating matters, Chen et al.'s research revealed that three out of four patients treated with quinidine showed no clinical improvement, and one patient experienced a recurrence of seizures despite a reduced frequency [79]. Additionally, the limitations of quinidine are not confined to its inconsistent efficacy; Mullen et al. pointed out its lack of effectiveness in ADNFLE and its dose-dependent cardiotoxicity, such as QT interval prolongation [75]. Similarly, Madaan et al. reported the first case in India of a pediatric patient failing quinidine treatment for refractory migrating partial epilepsy [50, 82]. Importantly, Abdelnour et al. observed an age-dependent response to quinidine in a group of eight patients, with complete effectiveness in children under 4 years old (*P* < 0.05) and no therapeutic benefit in older patients [83]. This variability highlights the complex factors influencing the response to quinidine, including the age at which treatment is initiated, the efficiency of drug penetration into the brain, the location of the mutation, and the presence of comorbid risk factors.

Beyond quinidine, other ion channel blockers such as bepredil and clofilium have been shown to inhibit Slack channels in vitro [84]. Recent advances in drug discovery have identified novel small-molecule inhibitors with promising preclinical profiles. For instance, Spitznagel et al. utilized a high-throughput thallium flux assay to characterize VU0606170, a potent inhibitor that suppressed wild-type K_Na_1.1 channels in HEK293 cells. Notably, this compound reduced neuronal excitability and firing rates in rat cortical neurons, suggesting its potential to counteract pathological hyperexcitability via selective K_Na_1.1 modulation [76]. In parallel, Griffin et al. employed a functional screening platform to identify a trifluoromethyl pyridyl analog (Compound 31), which exhibited in vivo efficacy by reducing seizure frequency and shortening inter-seizure intervals in a *KCNT1*-P924L mouse model [53]. Despite these advancements, the translation of ion channel blockers into clinical practice remains challenging, largely due to the intricate pathophysiology associated with *KCNT1*-related channel dysfunction. The distinct pathophysiology of *KCNT1*-related epilepsy requires specialized therapeutic strategies that go beyond conventional ASMs. Further clinical studies are necessary to validate the efficacy of these novel compounds.

### Ketogenic diet

The ketogenic diet (KD) is a nutritional intervention characterized by a high-fat (70–80%), very-low-carbohydrate (5–10%), and moderate-protein (10–20%) composition, requiring micronutrient supplementation tailored to individual needs [85, 86]. Its core mechanism involves the generation of ketone bodies (β-hydroxybutyrate and acetoacetate) through fatty acid β-oxidation, which replaces glucose as the primary energy source for the brain, thereby stabilizing mitochondrial ATP supply and modulating neuronal excitability [87]. Ketones suppress glutamatergic excitatory signaling, enhance GABAergic inhibitory transmission, and reduce the propagation of aberrant electrical activity, playing a pivotal role in managing refractory epilepsy associated with *KCNT1* gene mutations. The KD offers an an effective alternative therapy for patients who do not responded to ASM treatments and is generally considered safer and more effective than pharmacological treatments and surgical interventions. Nonetheless, there are limitations such as the possibility of nutrient deficiencies, gastrointestinal discomfort [88], and the risk of metabolic acidosis due to increased ketone levels, which require close monitoring and personalized management strategies [89]. In conclusion, while the KD can be a beneficial therapeutic option, its implementation requires a thorough evaluation of the patient’s unique requirements and circumstances.

### Gene therapy

Gene therapy is an innovative treatment approach under investigation for the management of seizures, specifically targeting epileptogenic regions to preserve surrounding healthy tissue and reduce the side effects of ASMs. This is especially significant for approximately one-third of the epilepsy patients who do not respond to standard medications [90]. Traditional pharmacological treatments often fail to yield satisfactory results. Although surgery is an alternative option for patients with clearly defined lesions or seizure foci, the complex nature of epileptic networks makes it challenging to fully localize and excise the epileptogenic zone [91].

Advances in technology have shed light on the genetic basis of various diseases, opening the door to a wide array of gene therapies. Antisense oligonucleotides (ASOs) are single-stranded nucleotide sequences that can bind to complementary RNA molecules, thereby regulating gene expression at the transcriptional or translational level [92]. Research by Burbano et al. [93] indicates that ASO-mediated gene silencing is a promising therapeutic approach for *KCNT1*-associated epilepsy, significantly reducing seizure frequency, improving abnormal behaviors, and increasing survival rates in animal models. ASOs are designed to target specific RNA sequences, modulate gene expression, and potentially decrease seizure occurrence. Their ability to cross the blood–brain barrier enables precise targeting of the central nervous system, enhancing their value in precision medicine. The customization of gene vectors for different conditions is essential for broadening the application of gene therapy, and the identification of appropriate therapeutic targets remains a critical challenge. Gene therapy requires thorough testing for safety and tolerability to ensure its effectiveness and to avert adverse reactions. Gene therapy could soon herald a new era of precision medicine in clinical practice.

## Conclusions

This study focuses on the role of Slack channels in modulating neuronal excitability in the central nervous system and explores how mutations in these channels lead to cognitive and motor deficits in individuals with severe, intractable epilepsy. Specifically, it underscores the challenges in diagnosing and managing *KCNT1*-related epilepsy due to the disorder’s complexity, which manifests in a variety of clinical presentations and inheritance modes. To develop effective treatments, it is crucial to comprehend the pathogenic mechanisms associated with *KCNT1* mutations. The research emphasizes the promise of enhanced management strategies, such as targeted gene therapy and the creation of new ASMs, that could arise from advancements in genetic screening and epilepsy genetics. Further study into the molecular basis of *KCNT1*-related epilepsy and the exploration of potential therapeutic interventions is needed to improve the quality of life for those affected.

## Data Availability

Availability of data and materials is not applicable in this study.

## Abbreviations

AP: Action potential
AHP: After-hyperpolarization
AHPs: After-hyperpolarizations
ADNFLE: Autosomal dominant nocturnal frontal lobe epilepsy
ASMs: Anti-seizure medications
ADHD: Attention deficit hyperactivity disorder
ASO: Antisense oligonucleotide
BLA: Basolateral amygdala
CNS: Central nervous system
CTD: C-terminal domain
DRG: Dorsal root ganglion
EIMFS: Epilepsy with migrating focal seizures
EOEE: Other early-onset epileptic encephalopathies
EEG: Electroencephalography
EEE: Early epileptic encephalopathy
EME: Early myoclonic encephalopathy
FMRP: Fragile X mental retardation protein
FXS: Fragile X syndrome
GOF: Gain of function
ILAE: International League against Epilepsy
ISs: Infantile spasms syndrome
K_Na_: Sodium-activated potassium channels
KD: Ketogenic diet
LGS: Lennox-Gastaut syndrome
MNTB: Medial nucleus of the trapezoid body
Nav: Voltage dependent sodium channel
NAD^+^: Niacinamide adenine dinucleotide
nAChR: Nicotinic acetylcholine receptor
OB: Olfactory bulb
OS: Ohtahara syndrome
PET: Positron Emission Tomography
RCK: K^+^ conductance regulatio
SHE: Sleep-related hypermotor epilepsy
VSD: Voltage-sensing domain
VANs: Vestibular afferent neurons
WS: West syndrome
